# BDC12-4.1 T-Cell Receptor Transgenic Insulin-Specific CD4 T Cells Are Resistant to *In Vitro* Differentiation into Functional Foxp3^+^ T Regulatory Cells

**DOI:** 10.1371/journal.pone.0112242

**Published:** 2014-11-13

**Authors:** Ghanashyam Sarikonda, Georgia Fousteri, Sowbarnika Sachithanantham, Jacqueline F. Miller, Amy Dave, Therese Juntti, Ken T. Coppieters, Matthias von Herrath

**Affiliations:** 1 Diabetes Center, La Jolla Institute for Allergy and Immunology, La Jolla, California, USA; 2 Type 1 Diabetes R&D center, Novo Nordisk Inc., Seattle, Washington, USA; McGill University Health Center, Canada

## Abstract

The infusion of ex vivo-expanded autologous T regulatory (Treg) cells is potentially an effective immunotherapeutic strategy against graft-versus-host disease (GvHD) and several autoimmune diseases, such as type 1 diabetes (T1D). However, *in vitro* differentiation of antigen-specific T cells into functional and stable Treg (iTreg) cells has proved challenging. As insulin is the major autoantigen leading to T1D, we tested the capacity of insulin-specific T-cell receptor (TCR) transgenic CD4^+^ T cells of the BDC12-4.1 clone to convert into Foxp3^+^ iTreg cells. We found that *in vitro* polarization toward Foxp3^+^ iTreg was effective with a majority (>70%) of expanded cells expressing Foxp3. However, adoptive transfer of Foxp3^+^ BDC12-4.1 cells did not prevent diabetes onset in immunocompetent NOD mice. Thus, *in vitro* polarization of insulin-specific BDC12-4.1 TCR transgenic CD4^+^ T cells toward Foxp3^+^ cells did not provide dominant tolerance in recipient mice. These results highlight the disconnect between an *in vitro* acquired Foxp3^+^ cell phenotype and its associated *in vivo* regulatory potential.

## Introduction

Type 1 diabetes (T1D) is a chronic autoimmune disease characterized by gradual destruction of insulin-producing beta cells in pancreatic islets. In the non-obese diabetic (NOD) mouse model of T1D, insulin is an essential autoantigen (reviewed in [1]) and mice with certain mutations in the insulin gene do not develop diabetes [2]. In NOD mice CD4^+^ T cell infiltration into islets can be detected as early as 3-4 weeks of age. However, disease onset appears later in life between 10-24 weeks of age suggesting that there are two phases of the disease, the initiation phase, characterized by monocyte infiltration, and the propagation phase, where CD4^+^ and CD8^+^ T effector (Teff) cells accumulate leading to loss of >80% beta cell mass, coinciding with disease onset. The majority of CD4^+^ T cells that infiltrate pancreas are insulin-specific [3], reacting against the 15-amino acid region 9-23 of the insulin B-chain (InsB:9-23) [4]. Despite such restricted T-cell receptor (TCR) reactivity, insulin specific CD4^+^ T cells exhibit diverse TCR-α/β chain usage [5]. Several insulin reactive T cell clones have been generated, some from the pancreas of prediabetic NOD mice (i.e., the BDC12-4.1 [5]) and some from the pancreatic lymph nodes (PLN) (i.e., the 2H6 [6]). While a significant proportion of the clones appear to be pathogenic, including the BDC12-4.1 clone, some, e.g. the 2H6 T cell clone, are protective.

The presence of InsB:9-23 reactive CD4^+^ T cells in the periphery of NOD mice has historically been attributed to incomplete negative thymic selection [7], [8]. It was recently shown that negative selection mechanisms per se are in fact not critically impaired in NOD mice [9] but rather that InsB:9-23-reactive CD4^+^ T cells escape selection due to limited presentation of peptide in the thymus due to low affinity binding mode of the peptide to the I-A^g7^ major histocompatibility molecule [10].

Two different TCR transgenic (Tg) mouse lines, BDC12-4.1 [11] and 2H6 [12], both specific for InsB:9-23 peptide were established independently. BDC12-4.1 TCR Tg mice develop spontaneous insulitis but no diabetes in F1 mice (FVB x NOD), whereas diabetes manifests in NOD.RAG^KO^ (backcross 1 generation) but with only 40% penetrance [11]. We recently described that both effector and Foxp3^+^ Treg cells are generated in the periphery of BDC12-4.1.RAG^KO^ mice, where the latter account for the reduced penetrance of T1D in this mouse line [13]. On the other hand, 2H6 Tg mice (2H6.NOD or 2H6.NOD.SCID) have almost no signs of insulitis and do not develop diabetes [12]. We initially set out to assess the permissiveness of insulin-specific T cells from both strains toward *in vitro* iTreg cell induction. We found that 2H6 T cells did not expand *in vitro* in an antigen specific manner. Whereas BDC12-4.1 T cells expanded upon antigen-specific activation and moreover differentiated into Foxp3^+^ Treg in *in vitro* polarizing conditions (iTreg), they did not protect from diabetes onset upon adoptive transfer in prediabetic NOD mice. This data shows that the demonstrated *in vivo* ability of BDC12-4.1 insulin-specific T cells to differentiate into functional Foxp3^+^ Treg occurs inefficiently under *in vitro* conditions, despite acquisition of key phenotypic markers.

## Results

### Insulin recognition by BDC12-4.1 and 2H6 TCR transgenic T-cells

**Figure 1:**
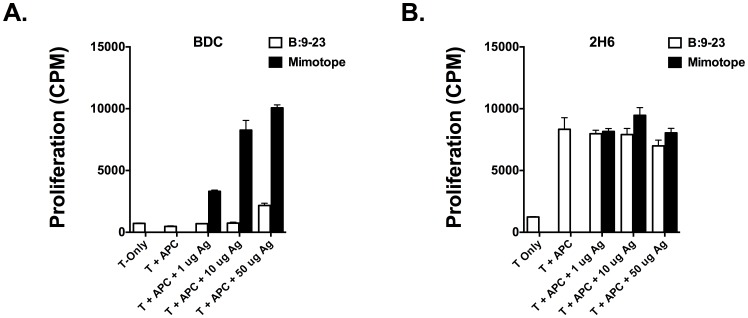
BDC12-4.1 TCR transgenic T cells exhibit robust insB:9-23-specific responses. CD4^+^ T cells purified from splenocytes of BDC12-4.1/RAG^KO^ or 2H6 mice were plated in triplicate, in round-bottom 96-well plates. 5–10×10^4^ T cells per well were incubated with an equal number of APCs for 48 hours. Native InsB:9-23 peptide (open bars) or R22E mimotope ([14]) of the InsB:9-23 peptide (filled bars) were used at the indicated concentration. T cells incubated alone or with only APCs (without added antigen) served to measure spontaneous proliferation. Mean and SEM values for average of the triplicate wells derived from pooled mice (n = 3) is shown.

To confirm the insulin recognition properties of the two InsB:9-23 specific T cells, we assessed their proliferative capacity in response to peptide stimulation. BDC12-4.1 T-cells exhibited robust proliferation in a dose-dependent manner (Fig. 1A). Exposure to R22E mimotope [14] of the InsB:9-23 peptide resulted in an enhanced dose-dependent proliferation. These results confirm the recognition of InsB:9-23 peptide as the cognate antigen for BDC12-4.1 T cells. On the other hand, we could not obtain convincing evidence of insulin-specific responses from 2H6 T cells. Addition of antigen presenting cells (APCs) to 2H6 TCR Tg cells in the absence of antigen resulted in non-specific proliferation. Such proliferation did not increase in magnitude upon the addition of the native InsB:9-23 peptide or the R22E mimotope (Fig. 1B). Further, although 2H6 T cells exhibited Ca^+2^ mobilization in response to InsB:9-23 stimulation, in some but not all of our experiments, (Fig. S1), they did not bind to I-A^g7^ tetramer (data not shown) loaded with the modified InsB:9-23 peptide [15]. Taken together, these results show that only T cells from the BDC12-4.1 clone respond to cognate antigen-specific stimulation and therefore we focused on generating iTregs only from BDC12-4.1 TCR Tg mice.

### BDC12-4.1 T cells expand in the periphery and display activated-memory and Treg phenotypes

**Figure 2:**
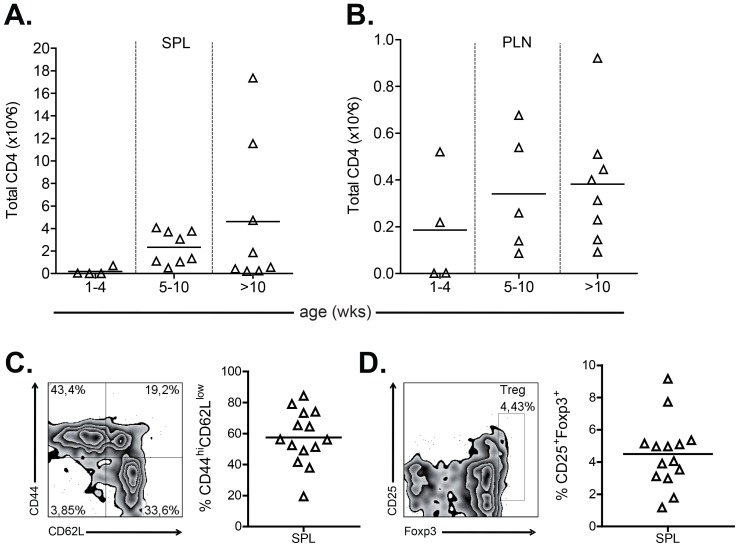
BDC12-4.1 T cells expand and display memory and Treg cell phenotypes in the periphery. (A and B) Age-associated increases in total CD4^+^ T cell numbers in the spleen and PLN of BDC12-4.1.RAG^KO^ mice. Splenocytes and PLN lymphocytes from BDC12-4.1.RAG^KO^ mice were analyzed at different ages by flow cytometry for the frequency of CD4^+^Vβ2^+^ T cells. The percentage of CD4^+^Vβ2^+^ cells was multiplied with the total number of cells isolated from the spleen or PLN to calculate the total number of CD4^+^ T cells. Trypan blue was used for the exclusion of dead cells prior to counting. (C and D) Representative FACS plots and data for activated T cells, CD44^hi^CD62L^low^, and Treg cells, CD25^+^Foxp3^+^, from the spleens of donor mice that were used to generate Foxp3^+^ Treg cells in vitro are shown.

Few BDC12-4.1 T cells escape thymic deletion [2] and undergo positive selection. Surprisingly, we found that CD4^+^CD8^+^ double positive thymocytes from BDC12-4.1 mice also express elevated levels of CD69 suggesting that some antigen-specific recognition is ongoing during positive selection (data not included). We previously showed that the majority of BDC12-4.1 TCR Tg cells acquire memory and Foxp3^+^ Treg phenotype in the spleen and PLN. Here, we also examined the number of total CD4^+^ T cells and found that in both spleen (Fig. 2A) and PLN (Fig. 2B) CD4^+^ T cells show a trend to expand with age. Prior to *in vitro* polarization into Foxp3 Treg, we evaluated the presence of CD44^hi^CD62L^low^ effector memory and Foxp3^+^ Treg cells in the spleens of the donor mice. As depicted in Fig. 2C and D, on average, 60% of CD4^+^ splenocytes displayed activated-memory phenotype and 5% expressed Foxp3. Whereas this data confirm our previous observations, it also shows that the starting CD4^+^ T cell population in the *in vitro* T cell cultures contains a great amount of already differentiated CD4^+^ T cells. Subsequently, we tested whether these cells can be polarized into functional Foxp3^+^ Treg cells.

### Cytokine production increases with age in BDC12-14.1 T cells

**Figure 3:**
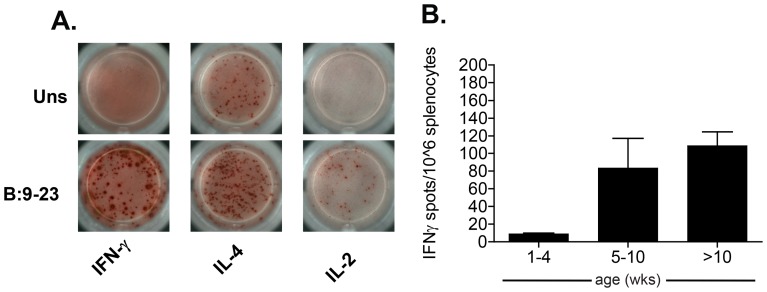
T cells with InsB:9-23 specific responses increase in frequencies with age. Total splenocytes from BDC12-4.1.RAG^KO^ mice were obtained at different ages. 250,000 splenocytes per well were incubated with or without native InsB:9-23 peptide in triplicates. Following 3-day incubation, IFN-γ, IL-2, and IL-4 production were determined by ELISpot assay. Representative images of cytokine production from BDC12-4.1.RAG^KO^ T-cells with (bottom row) or without antigen (top row) stimulation is shown in A. Background (media) subtracted IFN-γ spot numbers produced in response to antigen stimulation, from BDC12-4.1.RAG^KO^ mice of different age groups are shown on the Y-axis in B. Representative means ± SEM data from one of two independent experiments with n = 3 per each group with similar results are shown.

Before testing the plasticity of BDC12-4.1 T cells to differentiate into functional Foxp3^+^ Treg cells, we evaluated whether continuous antigen (insulin) exposure affects their functional properties. We found that BDC12-4.1 T-cells responded to InsB:9-23 stimulation by producing high amounts of IFN-γ, IL-4 and IL-2 cytokines (Fig. 3A), while the detection of other cytokines such as IL-10 and IL-17 was not as evident (data not shown). Interestingly, and in concordance with the rise in CD4^+^ T cell number cell with increasing age (Fig. 2), the number of cells producing IFN-γ in response to InsB:9-23 stimulation also increased (Fig. 3B). These results suggest that despite omnipresent *in vivo* antigen exposure, BDC12-4.1 T-cells remain functionally active and do not achieve an anergic state.

### *In vivo* polarized Foxp3^+^ cells from BDC12-4.1 mice do not protect recipient NOD mice from diabetes onset

**Figure 4:**
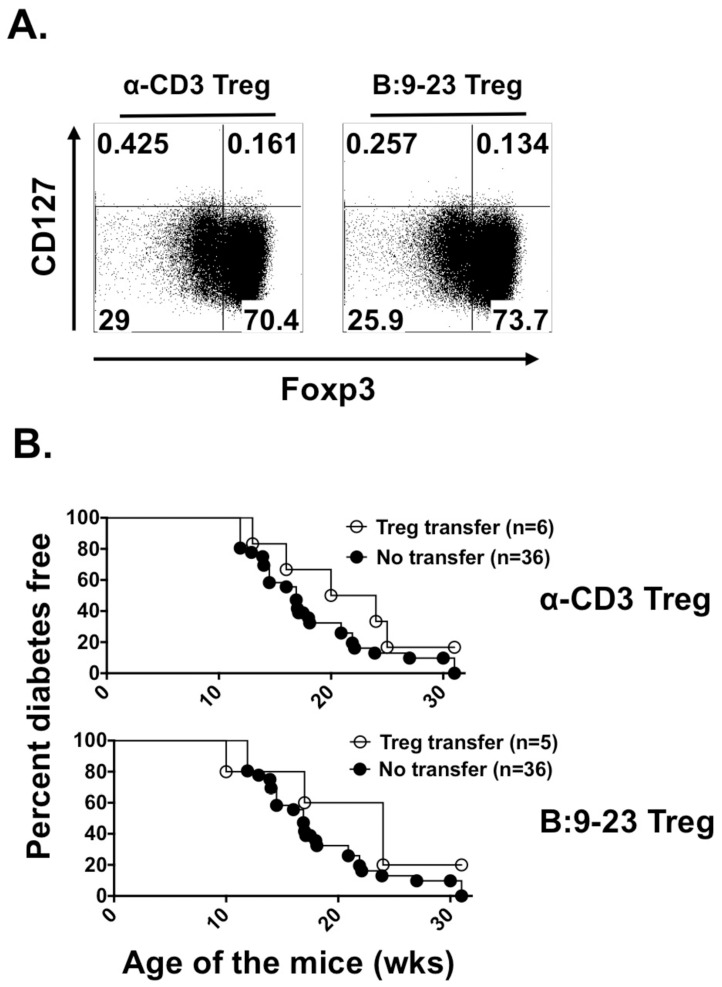
*In vitro* polarization induces Foxp3 expression in memory BDC12-4.1 T cells but does not endow *in vivo* regulatory functions. CD4^+^CD25^+^ cells from splenocytes of BDC12-4.1 mice polarized into Treg cells (A) *in vitro* in the presence of a-CD3 (left panel) or InsB:9-23 peptide (right panel). Expanded T cells were gated on CD4^+^CD25^+^ cells (not shown) and expression of CD127 and Foxp3 were determined. (B). 1×10^6^ Tregs polarized with anti-CD3 (left panel) or InsB:9-23 peptide (right panel) were adoptively transferred into prediabetic 8-wk old NOD mice and diabetes development was monitored. Unmanipulated NOD mice monitored on a regular basis in our animal colony served as negative controls with cumulative diabetes incidence of ∼90% by 27 wks of age. Representative results from one of three independent experiments are shown.

To assess whether pre-differentiated BDC12-4.1 T cells in peripheral tissues can be converted to protective iTregs, purified CD4^+^CD25^-^ T cells from BDC12-4.1 mice were cultured under *in vitro* Treg polarizing conditions with anti-CD3 or the cognate peptide antigen. In the presence of TGF-β and IL-2, BDC12-4.1 T cells readily differentiated into CD25^+^Foxp3^+^CD127^lo^ iTreg cells (Fig. 4A, phenotypically *bona-fide* Treg cells). To determine whether these *in vitro* polarized Foxp3^+^ cells could alter ongoing diabetogenic process we adoptively transferred them into fully immuno-competent 8–10 week old female NOD mice. Rather surprisingly, CD4^+^ BDC12-4.1 T cells expanded either in antigen-specific fashion (InsB:9-23) or with a nonspecific stimulation (a-CD3) failed to significantly protect recipient NOD mice from diabetes onset (Fig. 4B). Diabetes incidence in mice receiving Foxp3^+^ cells mirrored that of separate sets of control mice that serve to determine cumulative diabetes incidence in our NOD colony.

Among other polarizing conditions (Th1, Th2, Th17 or Tr1), BDC12-14.1 cells could be polarized toward Th17 and Tr1, evidenced by their ability to produce IL-17 and IL-10 respectively (Fig. S2A, B). Surprisingly, the expansion of BDC12-4.1 T cells toward Th17 or Tr1 (as compared to iTreg) was very limited, which compromised their use in further *in vivo* adoptive transfer experiments. After pooling results from two experiments where we generated sufficient Tr1 cells, few NOD mice were tested for diabetes protection, and a significant fraction of them was protected from diabetes (Fig. S2C). However, given the limited number of Tr1 cells that could be generated, we were unable to determine which factor(s) contributed to the differentiation of BDC12-4.1 cells into functional Tr1 cells.

## Discussion

Insulin, and in particular the 15 amino acid peptide of the B-chain 9-23, is an essential autoantigen in NOD mice (reviewed in [1]). While most InsB:9-23 reactive CD4^+^ T cell clones [5], like the BDC12-4.1 that was studied here are pathogenic [11], some, e.g., the 2H6 [12], are not. Understanding how recognition of insulin could endow pathogenic and/or regulatory roles to CD4^+^ T cells will help us understand how organ-specific autoimmunity develops and how it can be controlled. Here, by using BDC12-4.1 Tg mice, we found that: a) few InsB:9-23-specific CD4^+^ T cells escape negative selection and expand in the periphery, b) most peripheral BDC12-4.1 T cells are already differentiated into Teff or Treg, c) most cells that respond to InsB:9-23 peptide stimulation produce proinflammatory cytokines and d) under *in vitro* polarization conditions, peripheral BDC12-4.1 cells can be efficiently grown into phenotypic Treg (Foxp3^+^) cells, however, they do not protect recipient NOD mice from diabetes development upon adoptive transfer.

Recent evidence suggests that the InsB:9-23 peptide binds in at least two [16], [17], but likely three [15] different registers of I-A^g7^. Such differential binding and activation identifies two different set of T cells, type A and type B. Type A cells also recognize whole insulin processed and presented by thymic APCs and thus get deleted by negative selection. Type B cells do not recognize processed peptides escape thymic negative selection and in periphery they recognize InsB:9-23 peptide in register-2 leading to the generation of autoreactive T cells [16]. While this interesting observation explains how autoreactive T cells in NOD mice reach the periphery, it does not explain how two T cells reacting against the same InsB:9-23 peptide could develop into cells of two different fates, pathogenic (BDC12-14.1) and regulatory (2H6).

Although all CD4^+^ T cells express the same TCR in BDC12-4.1 mice, only a fraction becomes Treg *in vivo*. Consequently, a fraction of BDC12-4.1.RAG^KO^ mice are protected from diabetes, likely depending on the Teff: Treg ratio that is established *in vivo* [13]. In the present study, BDC12-4.1 T cells were induced to uniformly express Foxp3 *in vitro*, but failed to protect from diabetes. Thus, although one may be able to achieve a Treg phenotype *in vitro* over the course of days, other mechanisms probably act in vivo to lock in Tregs' full suppressive functionality. Existence of T-cells of multiple antigen specificities in recipient mice at the time of adoptive transfer (∼8-10 weeks of age) could contribute to the lack of protection upon adoptive transfer of Tregs of one antigen specificity, insB:9-23. And as such, Treg transfers into younger mice with a smaller T-cell repertoire or with lesser autoantibody presence/levels may provide a different result. Various other possibilities could also account for the lack of protection; perhaps the number of transferred cells was low, the infused iTreg cell population was functionally unstable or it was contaminated by effector T cells.

Multiple lines of evidence suggest that strength of the TCR signal involving the trimolecular complex of TCR-MHC-peptide can dictate the fate of the T cell. For instance, in a different T-cell model, a two-fold increase in the concentration of MHC-antigen can make a responding CD8^+^ T cell go from a non-dividing cell to a dividing cell [18] and similar dose responses have been described for CD4^+^T cells [19]. Further studies are needed to address whether the location, the type of the APC or the cytokine microenvironment are responsible for dictating the fate of a naïve T cell or whether its fate is already predetermined in the thymus. We found that iTreg cells generated from thymocytes, analogous to splenocytes, cannot transfer protection from T1D (data not shown). Given that these studies are very preliminary (only 4 NOD mice tested), additional experiments are necessary to determine how we can drive BDC12-4.1 TCR Tg CD4^+^ T cells toward a stable Foxp3^+^ regulatory fate. Further, the fate of the *in vitro* generated BDC12-4.1 Tregs i.e., whether they retain their Treg phenotype *in vivo* and its role in protection from T1D onset could be explored. As cell therapy with *in vitro* generated Treg cells moves closer to the clinic, [20], [21] it will be essential to determine whether it is feasible to alter the differentiation state of a T cell with an acquired functional phenotype.

In conclusion, we demonstrated that *in vitro* reprogramming of antigen-specific, self-reactive T cells into Foxp3-expressing, phenotypic Treg is possible. However, the resulting population was not endowed with functional suppressive capacity under our experimental conditions. Our data show that when pre-differentiated autoantigen-specific CD4^+^ T cells are used as a starting population, their differentiation conditions need to be optimized to enable therapeutic success.

## Materials and Methods

### Ethics statement

Mice were maintained in La Jolla Institute for Allergy and Immunology (LIAI) animal facility, under pathogen-free conditions following the LIAI institutional animal care and use committee (IACUC) guidelines for the use and care of laboratory animals. LIAI IACUC approved the experiments performed in this study.

### Mice

BDC12-4.1 TCR transgenic mice were kindly provided by Prof. George Eisenbarthwhile 2H6.RAG^+^ mice were provided by Dr. Li Wen's group. Presence of transgenic TCR-α was determined by PCR [2], while TCR-β presence was determined by FACS analysis of Vβ2 or Vβ14 expression. BDC12-4.1/RAG^+^ mice were crossed into RAG^KO^ background and F1 were intercrossed to obtain BDC12-4.1/RAG.^KO^ mice. RAG^KO^ status of offspring was confirmed by PCR analysis as well as the absence of B220 positive cells by FACS.

### Cytokine ELISPOT

Interferon (IFN)-γ, interleukin (IL)-4, IL-17, IL-10 and IL-2 were determined as previously described [13]. Spleen cells were prepared by homogenization in red blood cell lysis buffer and cultured at 2–4×10^5^ cells/well in triplicate. Cells were cultured in RPMI media supplemented with 10% FBS (Life technologies). InsB:9-23 peptide (50 µg/ml) used for stimulation was high performance liquid chromatography purified (>90%) and dissolved in sterile DMSO (SynPep, Dublin, CA). 3-amino-9-ethylcarbazole (AEC) substrate and Chromogen solution (BD Biosciences) were used as the detection method, and wells were counted and analyzed using the KS ELISPOT reader, software version 4.

### Proliferation assays

Purified CD4^+^ T cells from BDC12-4.1/RAG.^KO^ or 2H6 mice were plated in triplicate, in round-bottom 96-well plates, at 5–10×10^4^ T cells per well with an equal number of mitomycin C-treated T-depleted splenocytes (TDS) from a NOD mice as APCs. The cultures were pulsed with 1 µCi of [^3^H]-thymidine (2 Ci/mmol) on day 1 and harvested 16–24 h later using a Tomtec 96-well harvester. The samples were then counted using a Wallac Betaplate scintillation counter. The mean proliferation and SEM of triplicate cultures are shown.

### Flow cytometry

After a 2.4G2 blocking step, cells were stained for CD4, CD8, CD44, CD62L, CD69, B220, CD25, CD3, CD127, and Vβ2 or Vβ14 (eBioscience or Biolegend or BD-Pharmingen, San Diego, CA, USA). All antibody incubations were performed at 4°C for 30 minutes (isotype controls were included). Intracellular Foxp3 staining was performed using Foxp3 staining kit (eBioscience) and manufacturer's protocol. Intracellular cytokine staining was performed as described [13]. Cells were immediately acquired on a LSRII flow cytometer (BD Biosciences) and analyzed using the FlowJo software (Treestar).

### CD4^+^CD25^+^/CD25^-^ cell purification

From pooled splenocytes, CD4^+^CD25^+^ cells were purified using CD4^+^CD25^+^ Treg isolation kit (Miltenyi Biotec Inc., Auburn, CA) according to the manufacturer's protocol. CD4^+^CD25^-^ cells were obtained by collecting the flow through cells during positive selection of CD4^+^CD25^+^ cells. Confirmation of >80% purity was performed by FACS before *in vitro* culture.

### *In vitro* polarization and expansion of Foxp3^+^ cells

Purified CD4^+^CD25^+^ or CD4^+^CD25^-^ cells were cultured in 10% FBS/RPMI medium (Life Technologies). For activation, either plate-bound anti-CD3 (1 µg/ml) or InsB:9-23 peptide (50 µg/ml) was used for *in vitro* stimulation during culture period. For each polarization, the cells were cultured in media containing various supplements as follows; for Th1 polarization, IL-2 (50 U/ml), IL-12 (5 ng/ml), anti-IL-4 (10 µg/ml) were added, for Th2 polarization IL-2 (50 U/ml), IL-4 (5 ng/ml) and anti-IFN-γ (10 µg/ml) were added. For Th17, IL-1β (5 ng/ml), TGF-β (5 ng/ml), and IL-21 (5 ng/ml) were added. For Treg cultures, IL-2 (50 U/ml) and TGF-β (5 ng/ml), were added, and finally for Tr1 cultures, IL-2 (50 U/ml), dexamethasone (50 nM) and vitamin D3 (100 nM) were added. All the cytokines except IL-2 were obtained from Peprotech while IL-2 was obtained from R&D systems. The cells were cultured for 7-10 days with fresh culture media added at regular intervals of 2-3 days. At the end of culture period, *in vitro* polarized cells were rested for 3 days by removing the stimulating agent (anti-CD3 or InB:9-23) and culturing in supplemented media.

### Adoptive transfer of *in vitro* generated Treg cell lines

CD4^+^CD25^+^ cells were purified using Treg isolation kit (Miltenyi Biotech Inc,), resuspended in Ca^+2^/Mg^+2^ free PBS and 1×10^6^ cells/mouse were transferred into recipient NOD mice via i.v., tail vein injection. Unmanipulated NOD mice were used as controls for these experiments.

### Diabetes monitoring

Blood glucose levels were monitored weekly with OneTouch Ultra blood glucose monitoring system (LifeScan Inc., Milpitas, CA, USA) and mice were considered to be diabetic when two consecutive blood glucose values (BGV) measured above 250 mg/dl.

### Statistical analysis

Data are expressed as a mean ± SD. Statistical significance of the difference between means was determined using the 2-tailed Student's *t*-test using Graphpad PRISM software (Graphpad, San Diego, CA, USA). For diabetes incidence measurement, statistical significance was determined by Kaplan Meier analysis. *,p<0.05, **, p<0.01, ***, p<0.001.

## Supporting Information

Figure S1 **2H6 T cells mobilize calcium upon TCR stimulation.** Purified CD4^+^ cells from 2H6 mice were labeled with CFSE and Indo-1. TDS from a non-diabetic NOD mouse were incubated with 50 ug/mL InsB:9-23 peptide in the presence or absence of Glyphosine. 2H6 cells were stimulated with no antigen (APCs only, negative control) or a-CD3 cross-linking (positive control) or APCs incubated with InsB:9-23 peptide. Ionomycin stimulation was used as maximal strength positive control stimulation to induce Ca^+2^ flux from all cells. 2H6 cells were spun for 8 s at 8000 rpm in a microcentrifuge, right after the addition of the stimulation (APCs with or without the insB:9-23 peptide) followed by brief vortex and data acquisition on an LSR-II.
(TIF)

Figure S2 **BDC12-4.1 T cells can be polarized into Th17 and Tr1 cells.** CD4^+^ cells from BDC12-4.1 mice cultured *in vitro* under Th17 (A) or Tr1 (B) polarizing conditions. Such polarized cells were restimulated with B:9-23 and production of TNF-α, IFN-γ, IL-4, IL-17 or IL-10 was determined by ICCS. (C) 1×10^6^ Tr1's/mouse were adoptively transferred into prediabetic 8-wk old NOD mice and diabetes development was monitored.
(TIF)
