# Supplementary figures and images for: BDC12-4.1 T-Cell Receptor Transgenic Insulin-Specific CD4 T Cells Are Resistant to In Vitro Differentiation into Functional Foxp3+ T Regulatory Cells
Source: PLoS One. 2014 Nov 13;9(11):e112242. doi: 10.1371/journal.pone.0112242 (PMC4231041; doi:10.1371/journal.pone.0112242)

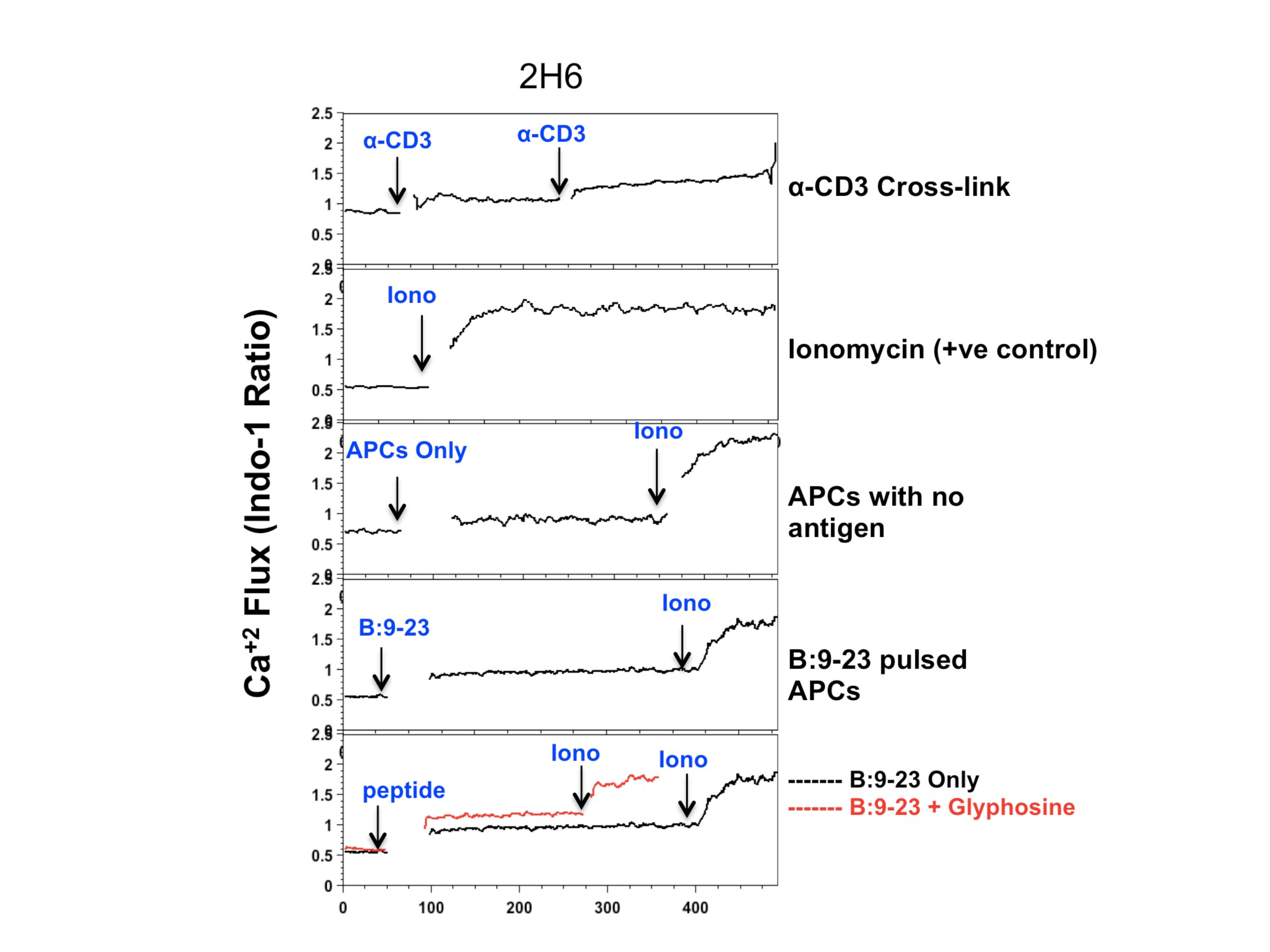

Supplement: Figure S1 — 2H6 T cells mobilize calcium upon TCR stimulation. Purified CD4+ cells from 2H6 mice were labeled with CFSE and Indo-1. TDS from a non-diabetic NOD mouse were incubated with 50 ug/mL InsB:9-23 peptide in the presence or absence of Glyphosine. 2H6 cells were stimulated with no antigen (APCs only, negative control) or a-CD3 cross-linking (positive control) or APCs incubated with InsB:9-23 peptide. Ionomycin stimulation was used as maximal strength positive control stimulation to induce Ca+2 flux from all cells. 2H6 cells were spun for 8 s at 8000 rpm in a microcentrifuge, right after the addition of the stimulation (APCs with or without the insB:9-23 peptide) followed by brief vortex and data acquisition on an LSR-II. (TIF) [file pone.0112242.s001.tif]

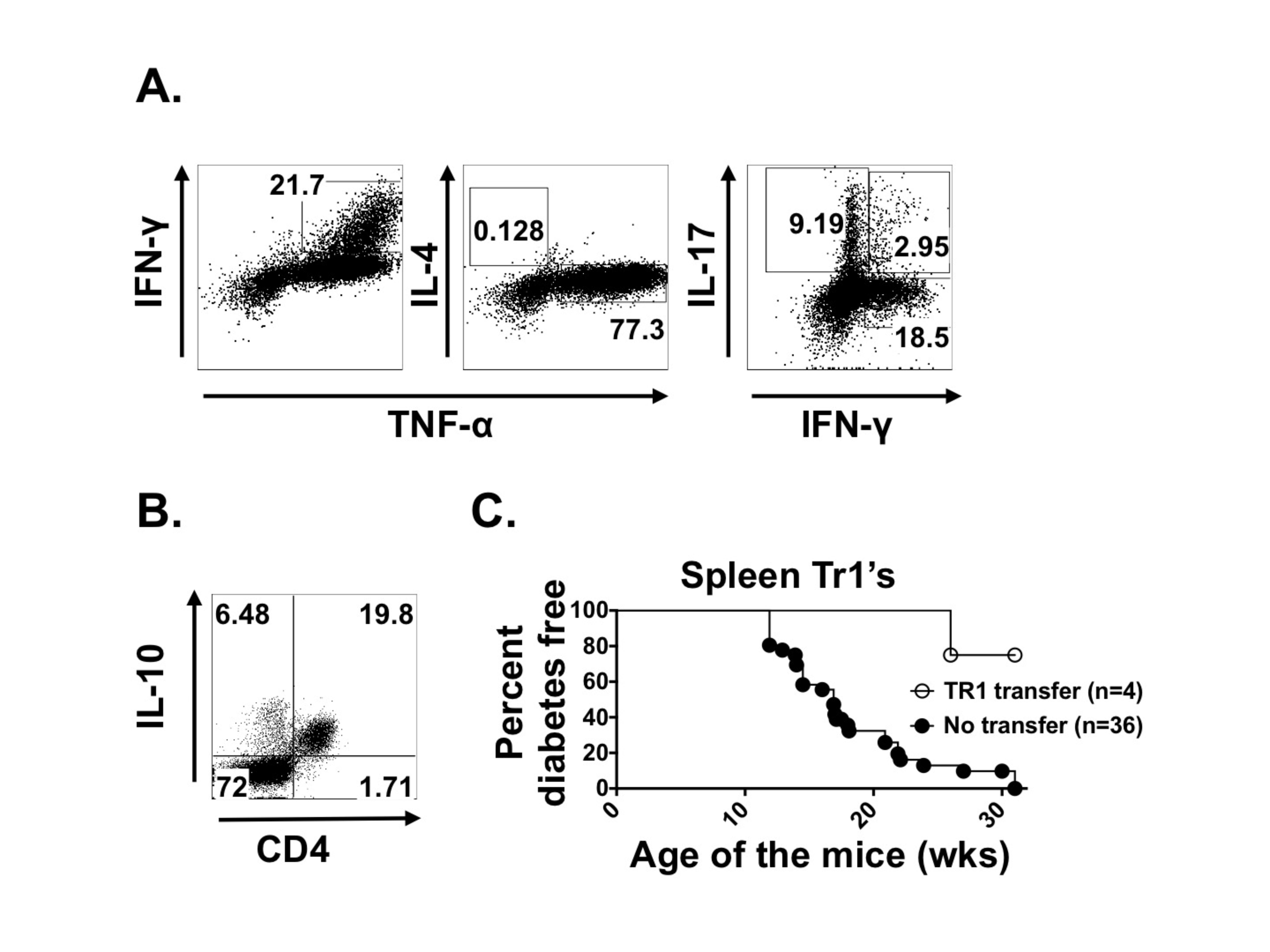

Supplement: Figure S2 — BDC12-4.1 T cells can be polarized into Th17 and Tr1 cells. CD4+ cells from BDC12-4.1 mice cultured in vitro under Th17 (A) or Tr1 (B) polarizing conditions. Such polarized cells were restimulated with B:9-23 and production of TNF-α, IFN-γ, IL-4, IL-17 or IL-10 was determined by ICCS. (C) 1×10^6^ Tr1's/mouse were adoptively transferred into prediabetic 8-wk old NOD mice and diabetes development was monitored. (TIF) [file pone.0112242.s002.tif]
